# Supplementary material for: Composite regulation of ERK activity dynamics underlying tumour-specific traits in the intestine
Source: Nat Commun. 2018 Jun 5;9:2174. doi: 10.1038/s41467-018-04527-8 (PMC5988836; doi:10.1038/s41467-018-04527-8)
Supplement: Supplementary file 1 — Supplementary Information [file 41467_2018_4527_MOESM1_ESM.pdf]

Composite regulation of ERK activity dynamics underlying tumour-specific traits in the intestine

Yu Muta et al.

Supplementary Information includes

Supplementary Figures: pages 2-13

Supplementary Table 1: page 14

Supplementary Table 2: page 15

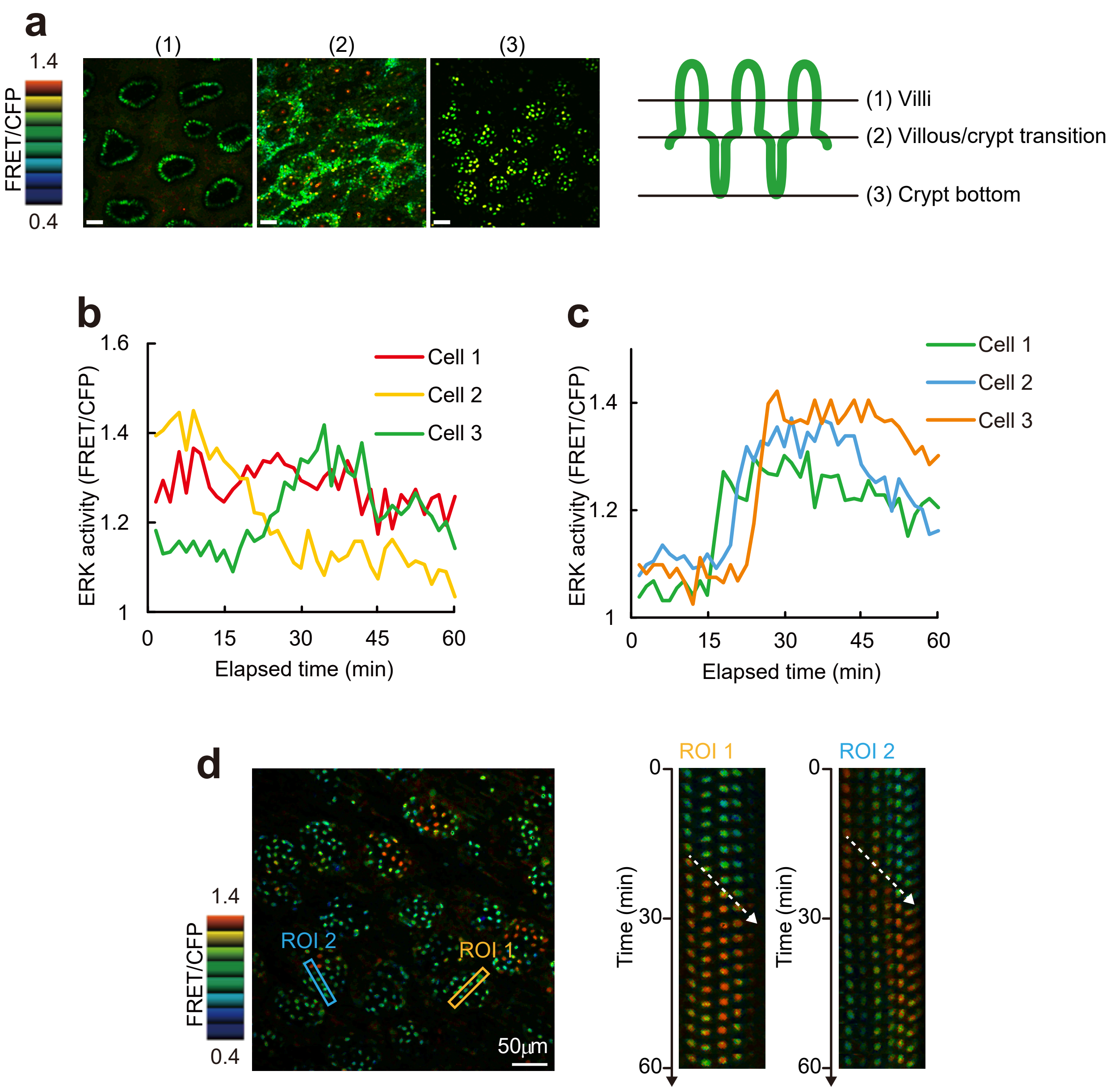

Supplementary Figure 1. ERK activity dynamics in the mouse small intestine. (**a**) In vivo imaging of the small intestine in EKAREV-NLS mice. The planes in each picture (1-3) correspond to schematic illustrations showing villi (1), transition between villi and crypts (2), and the crypt bottoms (3), respectively. Images are shown in the intensity-modulated display (IMD) mode. (**b, c**) The original time course data of ERK activity in the mouse intestinal epithelium before smoothing by the moving average. The data were analysed and used for Fig. 1h (**b**), and Fig. 1j (**c**). (**d**) Kymographs of in vivo imaging data showing the lateral propagation of ERK activity (right). Each graph was generated from the region of interest (ROI) indicated in the reference image (left). Scale bars, 50 μm.

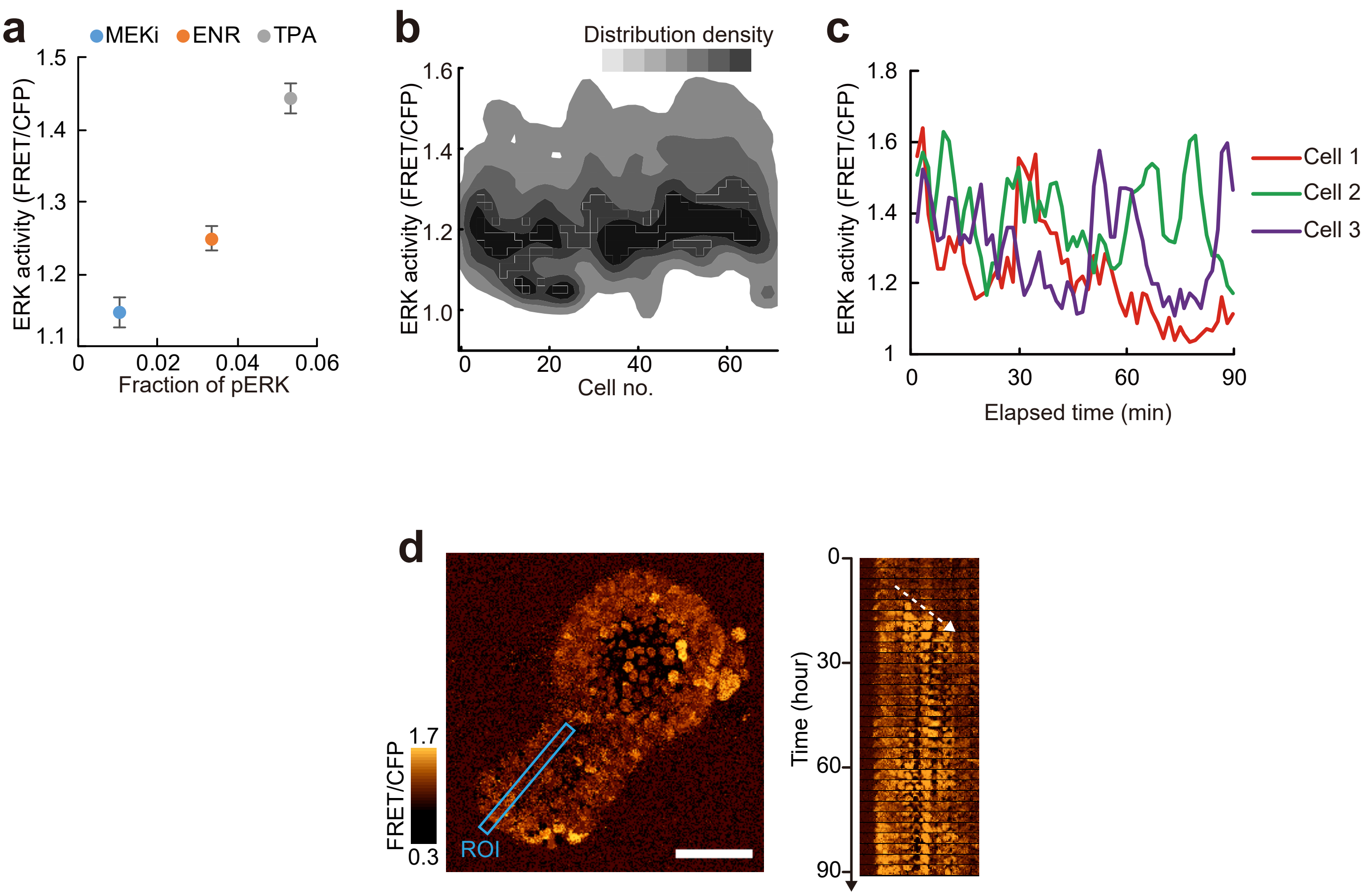

Supplementary Figure 2. Pulse like ERK activition and propagation in the intestinal organoids. (**a**) Organoids cultured in the ENR media were treated with vehicle (ENR), 200 nM of a MEK inhibitor, PD0325901 (MEKi), or 500 nM of TPA. Live imaging was performed 60 min after the treatment, and the FRET/CFP ratios under each condition were quantified. In parallel, organoids treated under the same condition were lysed with SDS sample buffer and subjected to immunoblotting with anti-ERK and anti-phosphorylated ERK (pERK) antibodies. The fraction of pERK/ERK was determined by comparing the relative amount of ERK and pERK to a reference sample whose pERK/ERK ratio was quantified based on the phosphorylation-dependent mobility shift of ERK in SDS-PAGE. (**b**) Distribution density of ERK activity in each cell. Darker colours indicate that the ERK activity was at the indicated value for a longer time (n = 71 cells). (**c**) The original time course data of ERK activity in an intestinal organoid before smoothing by the moving average. The data were analysed and used for Fig. 3c. (**d**) A kymograph of time lapse imaging of an intestinal organoid (right) showing the lateral propagation of ERK activity. A region of interest (ROI) used to make the kymograph is shown in the reference image (left). Error bars represent s.e.m. Scale bars, 50 µm.

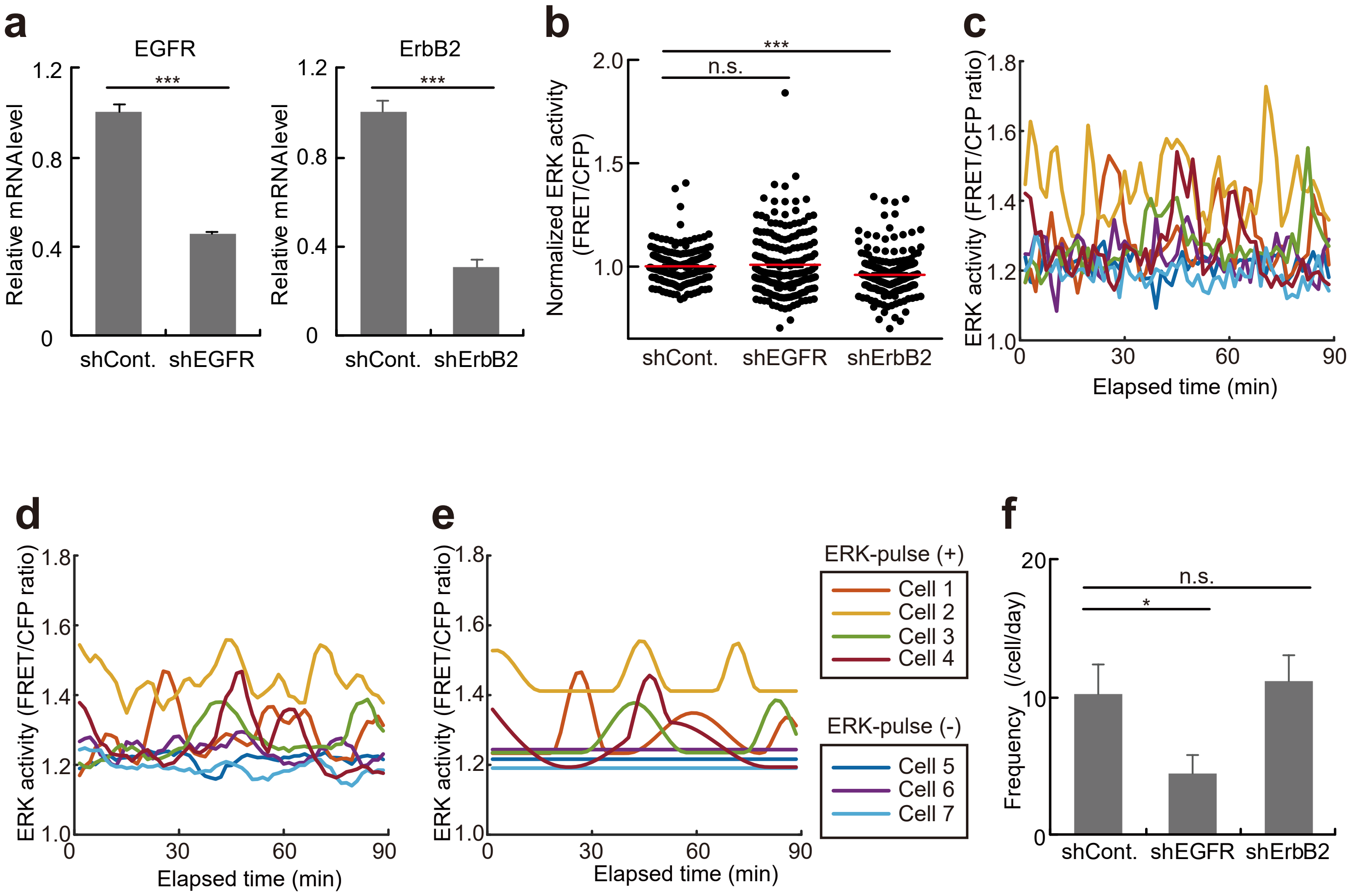

Supplementary Figure 3. ERK activity dynamnics regulated by EGFR and ErbB2 signalling. (**a**) The relative mRNA levels of EGFR (left) and ErbB2 (right) in MC-38 cells expressing a control shRNA (shCont.), or a shRNA for either EGFR (shEGFR) or ErbB2 (shErbB2) were analysed by RT-PCR (n = 5 samples). (**b**) Bee swarm plots of ERK activity in the organoids infected with lentiviruses expressing shCont., shEGFR, or shErbB2 (shCont.: n = 200, shEGFR: n = 190, shErbB2: n = 199 cells, pooled from 5 organoids). (**c-e**) Multi-peak and flat line fitting of the ERK activity data obtained from the 7 representative cells. The raw data (**c**) were smoothened by 6-min moving average (**d**), and fitted to flat lines or multi-peak functions (**e**). Cells whose data successfully fitted to multi-peak functions were defined as "ERK-pulse+" (Cell 1-4), and the others were defined as "ERK-pulse-" (Cell 5-7). (**f**) Quantification of the ERK activity pulses in organoids expressing shCont., shEGFR, or shErbB2. ERK activity data from each cell were processed as described for Figure 4e (shCont.: n = 50, shEGFR: n = 50, shErbB2: n = 49 cells). Red lines represent mean. Error bars represent s.e.m. Welch’ s t test (a) and Steel-Dwass test (b, f) were used for comparison. *P < 0.05, ** P < 0.001, ***P < 0.0001, n.s., not significant.

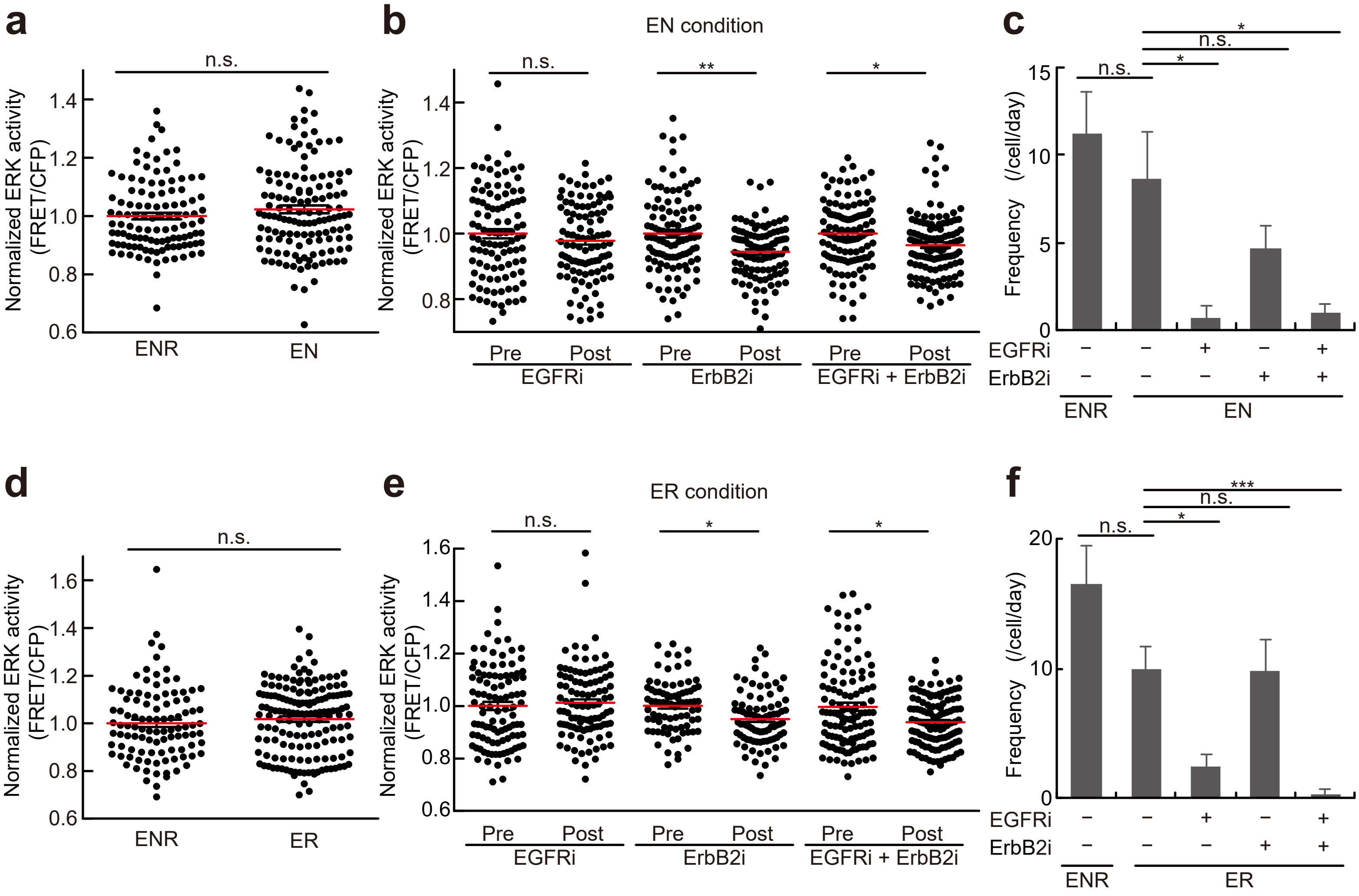

a
n.s.
Normalized ERK activity (FRET/CFP)
1.4
1.2
1.0
0.8
0.6
ENR
EN
b
EN condition
n.s.
**
*
Normalized ERK activity (FRET/CFP)
1.4
1.2
1.0
0.8
Pre
Post
Pre
Post
Pre
Post
EGFRi
ErbB2i
EGFRi + ErbB2i
c
*
n.s.
*
n.s.
Frequency (/cell/day)
15
10
5
0
EGFRi – – + – +
ErbB2i – – – + +
ENR
EN
d
n.s.
Normalized ERK activity (FRET/CFP)
1.6
1.4
1.2
1.0
0.8
0.6
ENR
ER
e
ER condition
n.s.
*
*
Normalized ERK activity (FRET/CFP)
1.6
1.4
1.2
1.0
0.8
0.6
Pre
Post
Pre
Post
Pre
Post
EGFRi
ErbB2i
EGFRi + ErbB2i
f
***
n.s.
*
n.s.
Frequency (/cell/day)
20
10
0
EGFRi – – + – +
ErbB2i – – – + +
ENR
ER

Supplementary Figure 4. Effects of R-spondin 1 or Noggin depletion in ERK activity dynamics. (**a-c**) Organoids were cultured in the R-spondin 1-depleted (EN) media for 24 h before imaging. (**a**) Bee swarm plots of ERK activity in organoids cultured in the ENR or EN media (n = 111 (ENR) and 133 (EN) cells pooled from 3 organoids). (**b, c**) Organoids cultured in the EN media were treated with 1 μM of an EGFR inhibitor (EGFRi), PD153035, and/or 10 μM of an ErbB2 inhibitor (ErbB2i), CP-724714. (**b**) Bee swarm plots of ERK activity before and after inhibitor treatment (EGFRi: n = 102, ErbB2i: n = 105, EGFRi + ErbB2i: n = 120 cells, pooled from 3 organoids). (**c**) Quantification of ERK activity pulses in organoids cultured in the ENR or EN media. Organoids were treated with EGFR and/or ErbB2 inhibitors, and then imaged for 90 min. ERK activity data from each cell were processed as described for Figure 4e (ENR/-/-: n = 40, EN/-/-: n = 35, EN/EGFRi/-: n = 48, EN/-/ ErbB2i: n = 31, EN/EGFRi/ ErbB2i: n = 50 cells). (**d-f**) Organoids were cultured in the Noggin-depleted (ER) media for 24 h before imaging. (**d**) Bee swarm plots of ERK activity in organoids cultured in the ENR or ER media (n = 102 (ENR) and 156 (ER) cells pooled from 3 organoids). (**e, f**) Organoids cultured in the ER media were treated with 1 μM of an EGFR inhibitor (EGFRi), PD153035, and/or 10 μM of an ErbB2 inhibitor (ErbB2i), CP-724714. (**e**) Bee swarm plots of ERK activity before and after inhibitor treatment (EGFRi: n = 102, ErbB2i: n = 83, EGFRi + ErbB2i: n = 15 cells, pooled from at least 2 organoids). (**f**) Quantification of ERK activity pulses in organoids cultured in the ENR or ER media. Organoids were treated with EGFR and/or ErbB2 inhibitors, and then imaged for 90 min. ERK activity data from each cell were processed as described for Figure 4e (ENR/-/-: n = 34, ER/-/-: n = 50, ER/EGFRi/-: n = 65, ER/-/ ErbB2i: n = 39, ER/EGFRi/ ErbB2i: n = 46 cells). Red lines represent mean. Error bars represent s.e.m. Mann–Whitney U-test (**a, b, d, e**) and Steel-Dwass test (**c, f**) were used for comparison. *P < 0.05, ** P < 0.001, ***P < 0.0001, n.s., not significant.

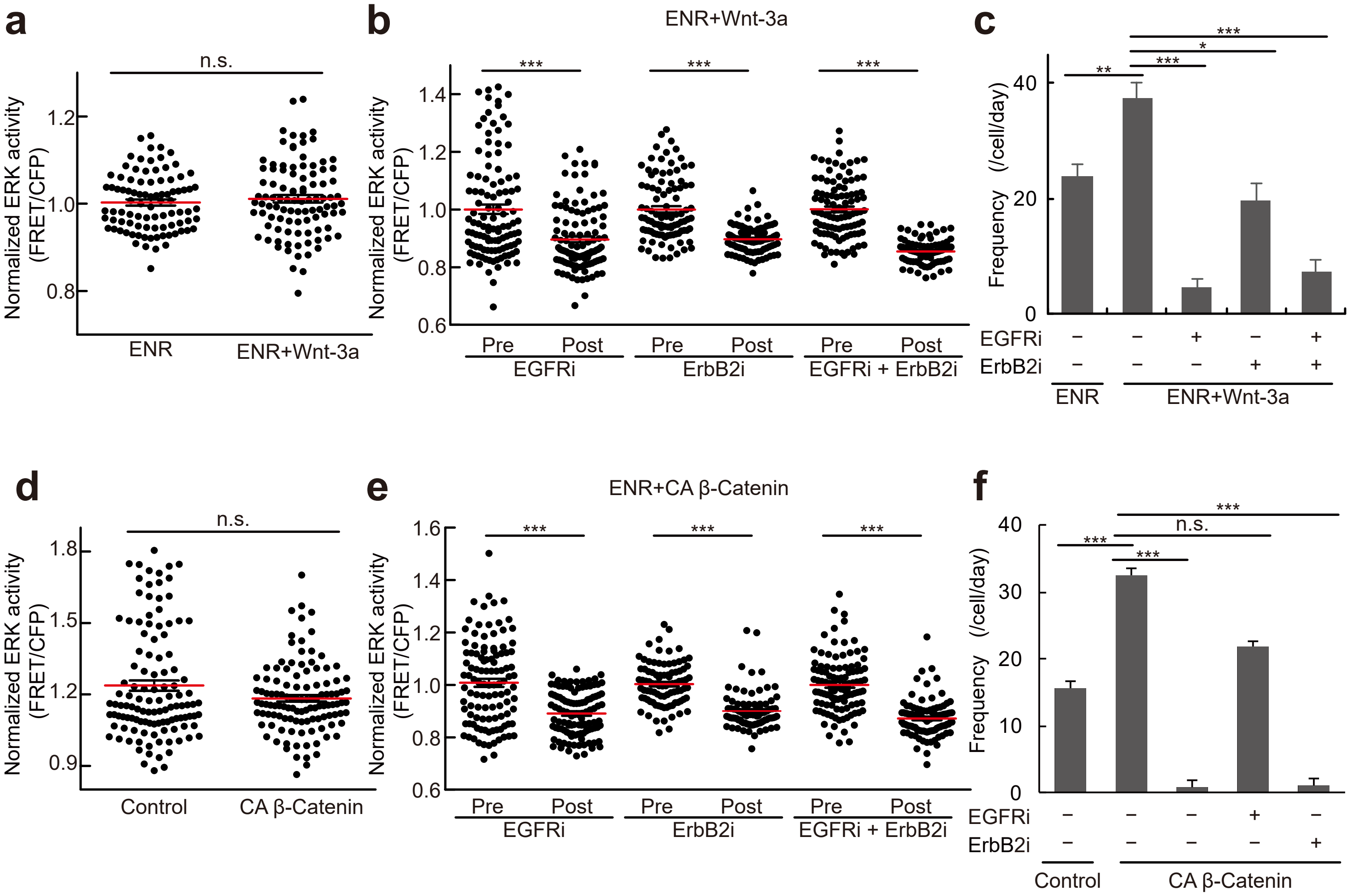

a
n.s.
Normalized ERK activity (FRET/CFP)
1.2
1.0
0.8
ENR
ENR+Wnt-3a
b
ENR+Wnt-3a
***
Normalized ERK activity (FRET/CFP)
1.4
1.2
1.0
0.8
0.6
Pre
Post
EGFRi
ErbB2i
EGFRi + ErbB2i
c
**
*
Frequency (/cell/day)
40
20
0
EGFRi
ErbB2i
ENR
ENR+Wnt-3a
d
n.s.
1.8
1.5
1.2
0.9
Control
CA β-Catenin
e
ENR+CA β-Catenin
1.6
f
n.s.
30
10

Supplementary Figure 5. Effects of Wnt signalling activation in ERK activity dynamics. (**a-c**) Organoids were cultured in ENR media supplemented with 100 ng ml^{-1} of Wnt3a (ENR + Wnt3a) for 24 h before imaging. (**a**) Bee swarm plots of ERK activity in organoids cultured in the ENR or ENR + Wnt3a media (n = 90 (ENR) and 98 (ENR + Wnt3a) cells pooled from 3 organoids). (**b, c**) Organoids cultured in the ENR + Wnt3a media were treated with 1 μM of an EGFR inhibitor (EGFRi), PD153035, and/or 10 μM of an ErbB2 inhibitor (ErbB2i), CP-724714. (**b**) Bee swarm plots of ERK activity before and after inhibitor treatment (EGFRi: n = 112, ErbB2i: n = 98, EGFRi + ErbB2i: n = 100 cells, pooled from 3 organoids). (**c**) Quantification of ERK activity pulses in organoids cultured in the ENR or ENR + Wnt3a media. Organoids were treated with EGFR and/or ErbB2 inhibitors, and then imaged for 90 min. ERK activity data from each cell were processed as described for Figure 4e (ENR/-/-: n = 74, Wnt3a/-/-: n = 32, Wnt3a/EGFRi/-: n = 41, Wnt3a/-/ ErbB2i: n = 35, Wnt3a/EGFRi/ ErbB2i: n = 37 cells). (**d**) Bee swarm plots of the ERK activity in the organoids infected with a control lentivirus (control) or a lentivirus expressing constitutively active form of β-Catenin (CA β-Catenin) (Control : n = 116, CA β-Catenin: n = 114 cells, from 4 organoids). (**e, f**) Organoids expressing CA β-Catenin were treated with 1 μM of an EGFR inhibitor (EGFRi), PD153035, and/or 10 μM of an ErbB2 inhibitor (ErbB2i), CP-724714. (**e**) Bee swarm plots of ERK activity before and after inhibitor treatment (EGFRi: n = 110, ErbB2i: n = 90, EGFRi + ErbB2i: n = 108 cells, pooled from at least 2 organoids). (**f**) Quantification of ERK activity pulses in control and CA β-Catenin organoids. Organoids were treated with EGFR and/or ErbB2 inhibitors, and then imaged for 90 min. ERK activity data from each cell were processed as described for Figure 4e (Control/-/-: n = 54, CA β-Catenin /-/-: n = 64, CA β-Catenin /EGFRi/-: n = 55, CA β-Catenin /-/ ErbB2i: n = 25, CA β-Catenin /EGFRi/ ErbB2i: n = 71 cells). Red lines represent mean. Error bars represent s.e.m. Mann–Whitney U-test (**a, b, d, e**) and Steel-Dwass test (**c, f**) were used for comparison. *P < 0.05, ** P < 0.001, ***P < 0.0001, n.s., not significant.

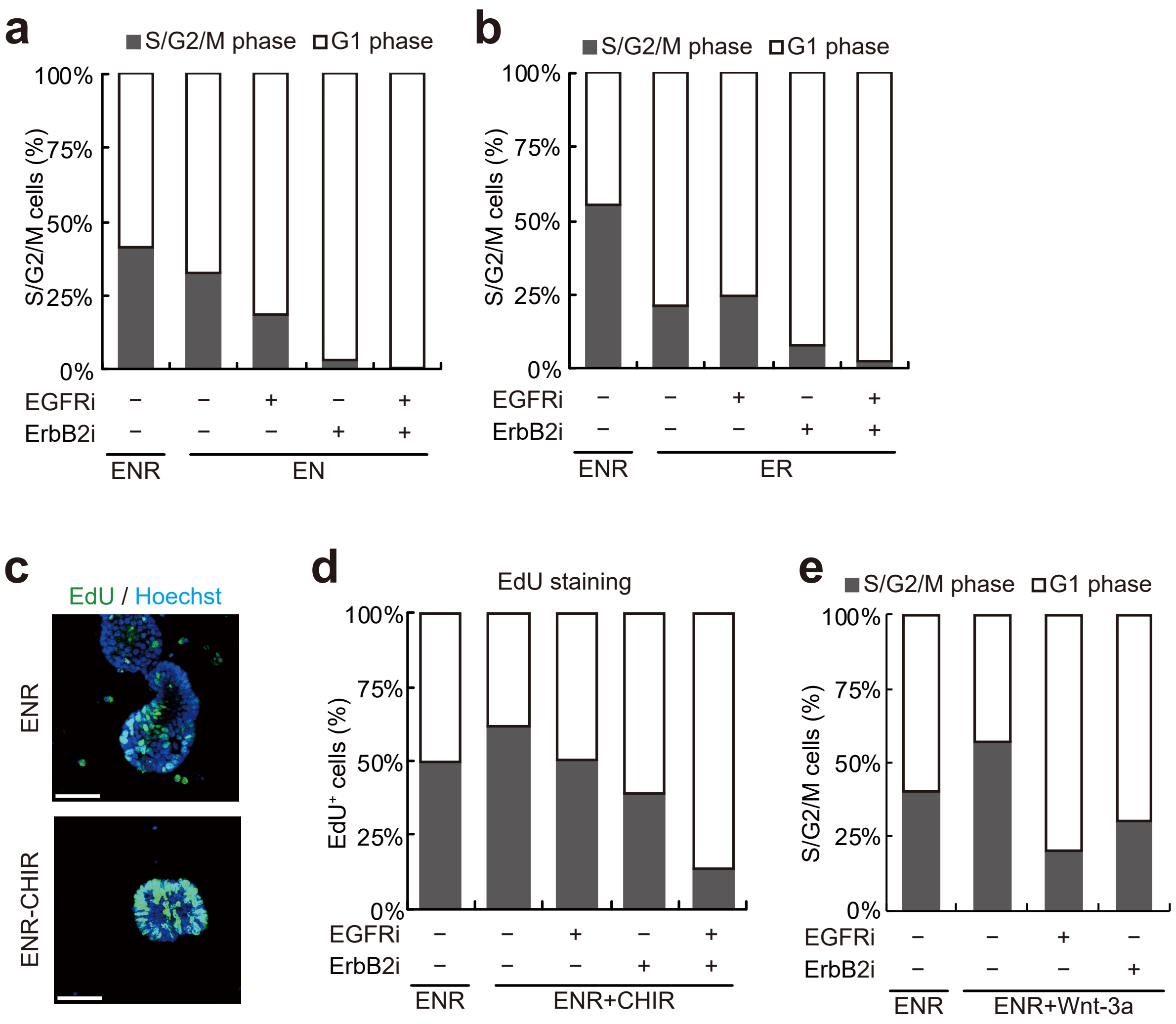

Supplementary Figure 6. Cell cycle analyses of organios under different conditions. (**a, b**) Proportion of cells in the S/G2/M phases in Fucci2a organoids cultured under different conditions. (**a**) Organoids were cultured in the R-spondin 1-depleted media (EN) supplemented with EGFRi and/or ErbB2i for 24 h. (ENR/-/-: n = 427, EN/-/-: n = 343, EN/EGFRi/-: n = 369, EN/-/ErbB2i: n = 249, and EN/EGFRi/ErbB2i/ n = 202 cells from 3 organoids) (**b**) Organoids were cultured in the Noggin-depleted media (ER) supplemented with EGFRi and/or ErbB2i for 24 h. (ENR/-/-: n = 226, ER/-/-: n = 312, ER/EGFRi/-: n = 242, ER/-/ErbB2i: n = 155, and ER/EGFRi/ErbB2i/ n = 252 cells from 3 organoids) (**c, d**) EdU staining of organoids treated with CHIR99021, an EGFR inhibitor, and/or an ErbB2 inhibitor for 24 h. Representative images (**c**) and proportion of EdU+ cells under different conditions (**d**) (ENR/-/-: n = 499, CHIR/-/-: n = 482, CHIR/EGFRi/-: n = 259, CHIR/-/ErbB2i: n = 267, and CHIR/EGFRi/ErbB2i/ n = 245 cells from more than 3 organoids). (**e**) Fucci2a organoids were treated with Wnt-3a, EGFRi, and/or ErbB2i for 24 h. Proportion of cells in the S/G2/M phases is shown. (ENR/-/-: n = 292, Wnt3a/-/-: n = 290, Wnt3a/EGFRi/-: n = 196, Wnt3a/-/ErbB2i: n = 240 cells from 3 organoids). Scale bars, 50 µm.

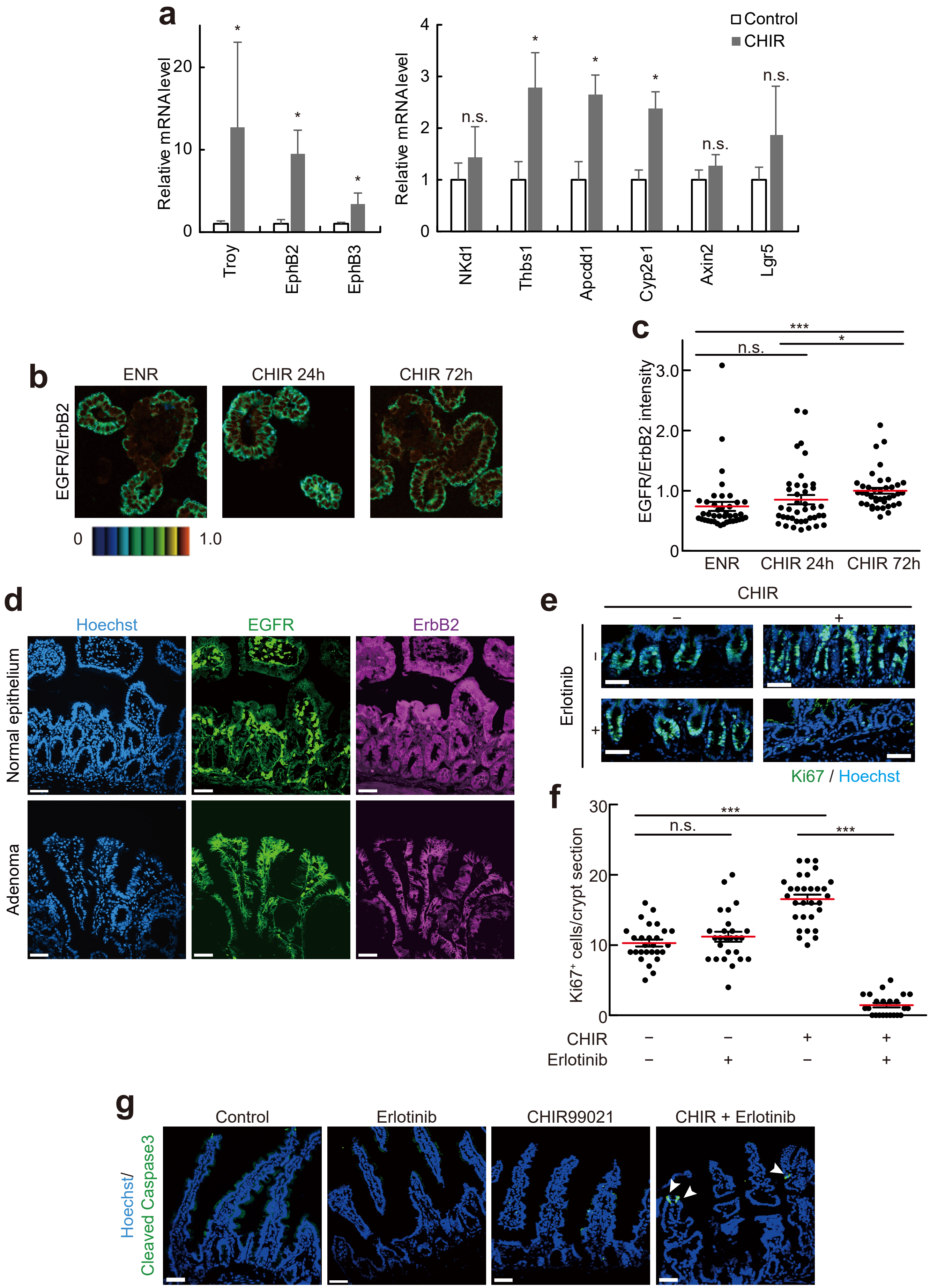
a
Relative mRNA level
20
10
0
Troy
EphB2
EphB3
*
Control
CHIR
4
3
2
1
n.s.
NKd1
Thbs1
Apcdd1
Cyp2e1
Axin2
Lgr5
b
ENR
CHIR 24h
CHIR 72h
EGFR/ErbB2
0
1.0
c
EGFR/ErbB2 intensity
3.0
2.0
1.0
***
n.s.
ENR
CHIR 24h
CHIR 72h
d
Hoechst
EGFR
ErbB2
Normal epithelium
Adenoma
e
CHIR
–
+
Erlotinib
Ki67 / Hoechst
f
Ki67⁺ cells/crypt section
30
20
10
0
CHIR
Erlotinib
g
Control
Erlotinib
CHIR99021
CHIR + Erlotinib
Hoechst/
Cleaved Caspase3

Supplementary Figure 7. Analyses of adenomas and CHIR99021 treatment in vivo. (**a**) The relative mRNA levels of the Wnt-target genes in the isolated small intestinal epithelial cells of control and CHIR99021-treated mice (CHIR) were analysed by RT-PCR. Mice were injected with vehicle or 20 mg kg^{-1} of CHIR99021 for 3days (n = 6 mice respectively). (**b, c**) Immunofluorescence staining with anti-EGFR and anti-ErbB2 antibodies of intestinal organoids treated with 5 μM of CHIR99021 for different duration; 0h (ENR), 24 h (CHIR 24h) and 72 h (CHIR 72h). The ratio of the EGFR and ErbB2 staining intensity in the IMD mode (**b**) and the bee swarm plots of the EGFR/ErbB2 staining intensity ratio (**c**) are shown (n = 40 cells pooled from 3 organoids). (**d**) Immunofluorescence staining of an adenoma and the normal small intestine of ApcΔ716 mice with anti-EGFR and anti-ErbB2 antibodies. The nuclei of cells were co-stained with Hoechst. Note that strong staining of cells under the submucosal layer results from non-specific binding of the secondary antibodies used here (anti-mouse IgG) to immune cells. (**e**) Immunofluorescence staining of the small intestine of CHIR99021- and/or erlotinib-treated mice with an anti-Ki67 antibody. The nuclei of cells were co-stained with Hoechst. (**f**) Quantification of Ki67+ cells per crypt section (-/-: n = 27, CHIR/-: n = 30, -/Erlotinib: n = 26, and CHIR/Erlotinib: n = 23 crypts). (**g**) Immunofluorescence staining of the small intestine of CHIR99021- and/or erlotinib-treated mice with an anti-cleaved caspase3 antibody. Mice were injected with vehicle, 20 mg kg^{-1} of CHIR99021, and/or 100 mg kg^{-1} of erlotinib for 3 days. Apoptotic cells were indicated by white arrowheads. Scale bars, 50 μm. Red lines represent mean. Error bars represent s.e.m. Mann–Whitney U-test (**a**), and Steel-Dwass test (**c, f**) were used for comparison. *$P < 0.05$, ** $P < 0.001$, ***$P < 0.0001$, n.s., not significant.

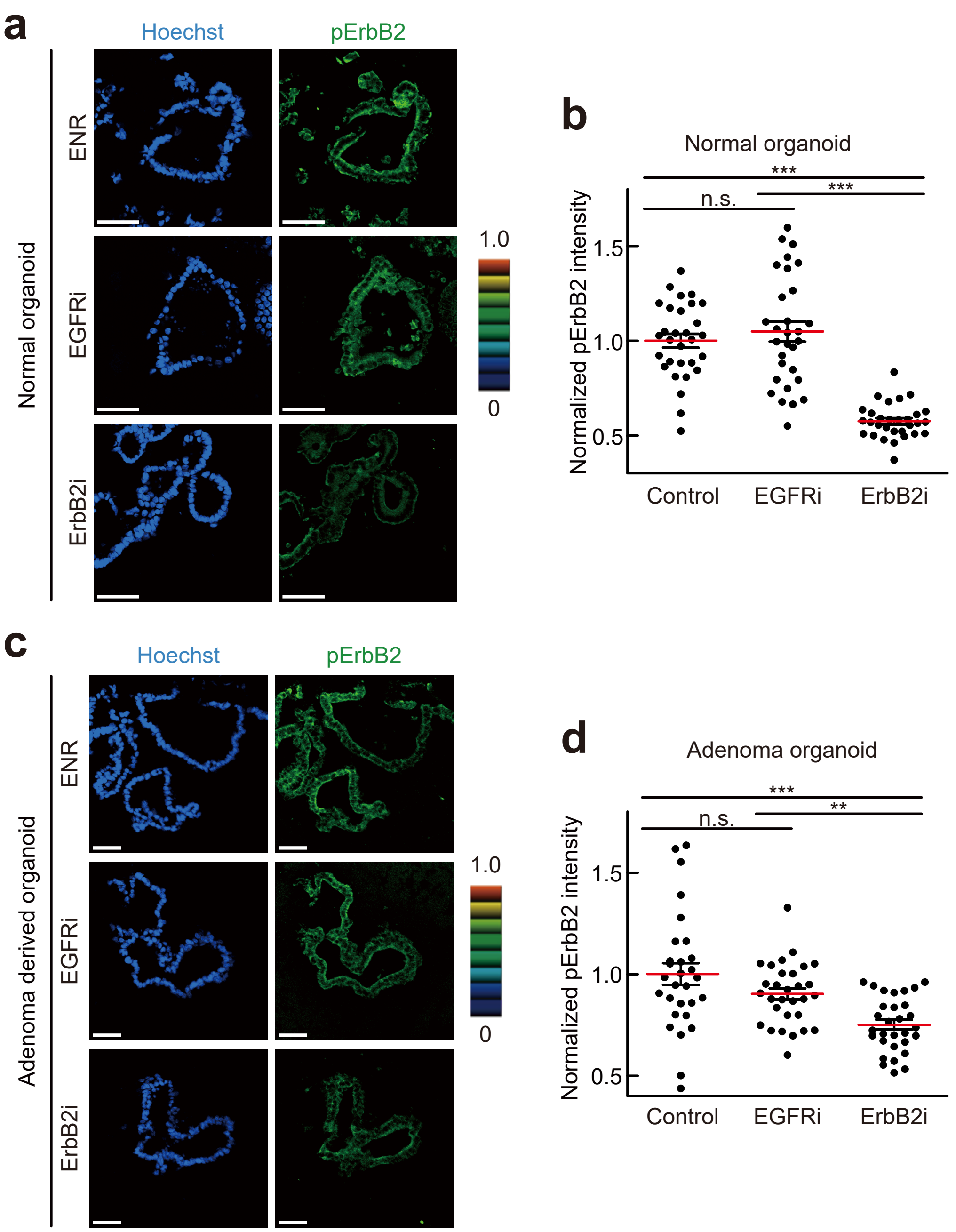

Supplementary Figure 8. Immunofluorescence staining of normal and adenoma organoids with anti-phosphorylated ErbB2. (**a, b**) Normal organoids were treated with either EGFRi or ErbB2i for 1 h, and immunofluorescence staining with an anti-phosphorylated ErbB2 (pErbB2) antibody was performed. The nuclei of cells were co-stained with Hoechst. (**b**) Bee swarm plots of pErbB2 staining intensity under each condition (n = 30 cells pooled from 2 organoids). (**c, d**) Adenoma-derived organoids were treated with either EGFRi or ErbB2i for 1 h, and immunofluorescence staining with an anti-pErbB2 antibody was performed. The nuclei of cells were co-stained with Hoechst. (**d**) Bee swarm plots of pErbB2 staining intensity under each condition (Control: n = 29, EGFRi: n = 30, and ErbB2i: n = 30 cells). Scale bars, 50 μm. Red lines represent mean. Error bars represent s.e.m. Steel-Dwass test (**b, d**) was used for comparison. *$P < 0.05$, ** $P < 0.001$, ***$P < 0.0001$, n.s., not significant.

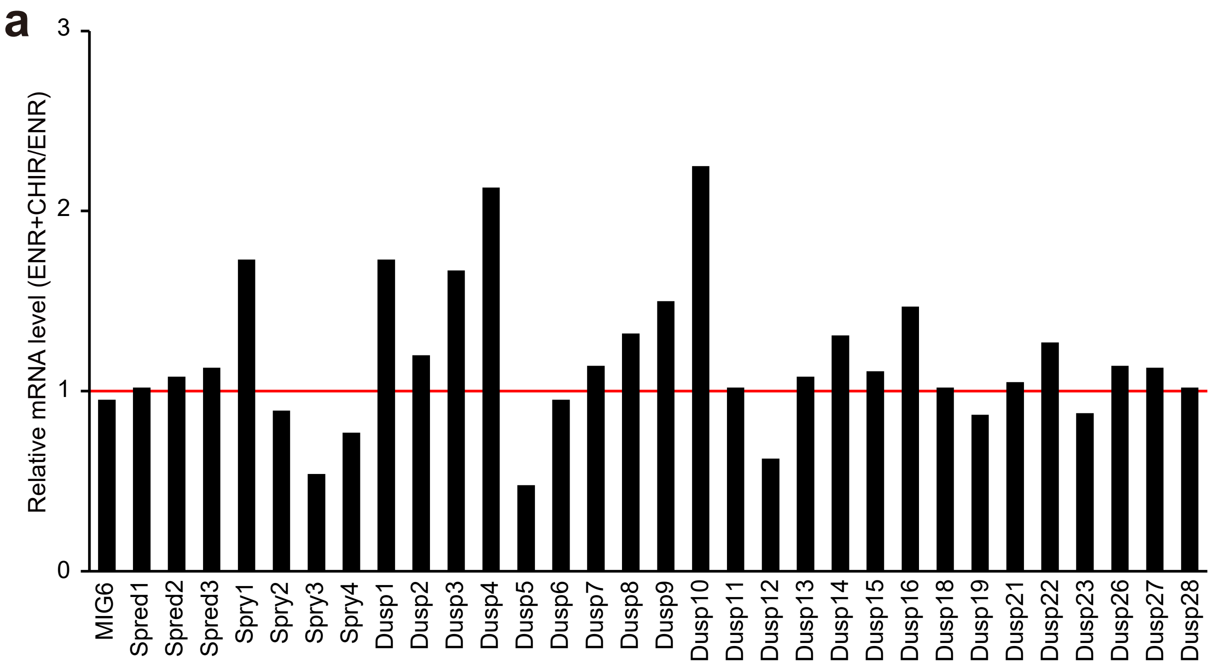

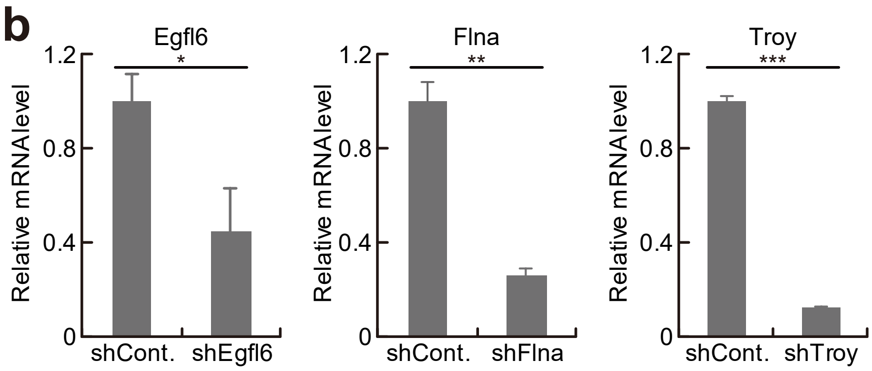

Supplementary Figure 9. Wnt signalling mediated EGFR regulators. (**a**) The relative mRNA levels of indicated genes were obtained from microarray analysis. (**b**) The relative mRNA levels of Egfl6 (left), Flna (middle), or Troy (right) in organoids expressing a control shRNA (shCont.), or a shRNA for Egfl6 (shEgfl6), Flna (shFlna), or Troy (shTroy) were analysed by RT-PCR (n = 5 samples respectively). Red lines represent mean. Error bars represent s.e.m. Welch' s t test (**b**) was used for comparison. *P < 0.05, ** P < 0.001, ***P < 0.0001, n.s., not significant.

Supplementary Table 1. Gene sets enriched in the CHIR99021-dependent upregulated or downregulated genes

Upregulated gene sets

Gene set name	# Genes in gene set (K)	Description	# Genes in overlap (k)	k/K	p-value	FDR q-value
SANSOM_WNT_PATHWAY_REQUIRE_MYC	58	Wnt target genes up-regulated after Cre-lox knockout of APC [GeneID=324] in the small intestine that require functional MYC [GeneID=4609].	9	0.1552	2.59E-16	4.61E-12
MEISSNER_BRAIN_HCP_WITH_H3K4ME3_AND_H3K27ME3	1069	Genes with high-CpG-density promoters (HCP) bearing histone H3 dimethylation at K4 (H3K4me2) and trimethylation at K27 (H3K27me3) in brain.	19	0.0178	1.64E-15	1.14E-11
PASINI_SUZ12_TARGETS_DN	315	Genes down-regulated in ES (embryonic stem cells) with defficient SUZ12 [GeneID=23512].	13	0.0413	1.92E-15	1.14E-11
HALLMARK_EPITHELIAL_MESENCHYMAL_TRANSITION	200	Genes defining epithelial-mesenchymal transition, as in wound healing, fibrosis and metastasis.	11	0.055	1.39E-14	6.18E-11
GO_LOCOMOTION	1114	Self-propelled movement of a cell or organism from one location to another.	18	0.0162	5.16E-14	1.84E-10

Downregulated gene sets

Gene set name	# Genes in gene set (K)	Description	# Genes in overlap (k)	k/K	p-value	FDR q-value
SANSOM_APC_TARGETS_DN	366	Top genes down-regulated at day 5 of Cre-Lox induced APC [GeneID=324] knockout in the intestine.	52	0.1421	1.58E-61	2.81E-57
FEVR_CTNNB1_TARGETS_UP	682	Genes up-regulated in intestinal crypt cells upon deletion of CTNNB1 [GeneID=1499].	53	0.0777	1.55E-48	1.38E-44
GO_SMALL_MOLECULE_METABOLIC_PROCESS	1767	The chemical reactions and pathways involving small molecules, any low molecular weight, monomeric, non-encoded molecule.	66	0.0374	1.07E-40	6.35E-37
REACTOME_BIOLOGICAL_OXIDATIONS	139	Genes involved in Biological oxidations	27	0.1942	6.51E-36	2.90E-32
GO_LIPID_METABOLIC_PROCESS	1158	The chemical reactions and pathways involving lipids, compounds soluble in an organic solvent but not, or sparingly, in an aqueous solvent. Includes fatty acids; neutral fats, other fatty-acid esters, and soaps; long-chain (fatty) alcohols and waxes; sphingoids and other long-chain bases; glycolipids, phospholipids and sphingolipids; and carotenes, polyprenols, sterols, terpenes and other isoprenoids.	52	0.0449	1.38E-35	4.93E-32

Supplementary Table 2. Target sequences for shRNAs

shRNA name	Target sequence (5'-3')
shFlna	AAGCTGGAACCTATAGCCTTA
shEgfl6	AAGAATATTGGCCGATTGAAA
shTroy	GGGAATGTTTCAGAATCTACT
shEGFR	ACCGAAATTTGTGCTACGCAA
shErbB2	AGGAGATCACAGGTTACCTAT
