# Supplementary material for: Composite regulation of ERK activity dynamics underlying tumour-specific traits in the intestine
Source: Nat Commun. 2018 Jun 5;9:2174. doi: 10.1038/s41467-018-04527-8 (PMC5988836; doi:10.1038/s41467-018-04527-8)
Supplement: Supplementary file 3 — Description of Additional Supplementary Files [file 41467_2018_4527_MOESM3_ESM.pdf]

## Description of Additional Supplementary Files

**File Name: Supplementary Movie 1**
**Description:** In vivo imaging of the mouse small intestine expressing FRET biosensor for ERK. This movie shows the spontaneous firing and propagation of ERK activity. Maximum projection of 16 z-sections spanning a total of 30 μm. Scale bars, 50 μm. Time shown in minutes:seconds.

**File Name: Supplementary Movie 2**
**Description:** Spontaneous ERK activation in an intestinal organoid. Maximum projection of 30 z-sections spanning a total of 38 μm. Scale bars, 50 μm. Time shown in minutes:seconds.

**File Name: Supplementary Movie 3**
**Description:** Lateral propagation of ERK activity in an intestinal organoid. Organoids were cultured in EGF-starved condition for 24 h before the imaging. Maximum projection of 28 z-sections spanning a total of 108 μm. Scale bars, 50 μm. Time shown in minutes:seconds.

**File Name: Supplementary Data 1**
**Description:** A list of CHIR99021-dependent upregulated and downregulated genes. Genes, whose protein products have been shown to regulate EGFR functions, are highlighted in orange.
